# Dermoscopy, Reflectance Confocal Microscopy and Optical Coherence Tomography Features of Acne: A Systematic Review

**DOI:** 10.3390/jcm11071783

**Published:** 2022-03-24

**Authors:** Antonio Alma, Alberto Sticchi, Camilla Chello, Stefania Guida, Francesca Farnetani, Johanna Chester, Vincenzo Bettoli, Giovanni Pellacani, Marco Manfredini

**Affiliations:** 1 Dermatology Unit, Department of Surgical, Medical, Dental and Morphological Sciences with Interest in Transplant, Oncological and Regenerative Medicine, University of Modena and Reggio Emilia, 41124 Modena, Italy; antonioalma@virgilio.it (A.A.); albi.sticchi@yahoo.it (A.S.); camilla.chello@gmail.com (C.C.); steguida@unimore.it (S.G.); francesca.farnetani@unimore.it (F.F.); johanna.chester@gmail.com (J.C.); pellacani.giovanni@gmail.com (G.P.); 2 Section of Dermatology and Infectious Diseases, Department of Medical Sciences, University of Ferrara, 44124 Ferrara, Italy; vincenzo.bettoli@gmail.com; 3 Department of Clinical Internal, Anesthesiological and Cardiovascular Sciences, Dermatology Clinic, Sapienza University of Rome, 00185 Rome, Italy

**Keywords:** acne, reflectance confocal microcopy, optical coherence tomography, non-invasive imaging, therapy, grading

## Abstract

Noninvasive imaging techniques have recently outlined precise microscopic features of acne elementary lesions and accurate quantifications for disease severity staging and therapeutical efficacy follow-up. The aim of this review is to systematically describe current applications of dermoscopy, reflectance confocal microscopy (RCM), and optical coherence tomography (OCT) in acne vulgaris assessment and management. The study followed the Preferred Reporting Items for Systematic Reviews and Meta-analyses (PRISMA) guidelines. We included studies conducted on human subjects with elementary lesions of acne vulgaris, reporting assessment of the lesions with dermoscopy, RCM, and/or OCT. At present there are few large studies regarding acne and noninvasive imaging techniques, representing the main limitation of this review. Clinical examination represents the first line in acne diagnosis and treatment. However, dermoscopy, RCM, and OCT are further tools that can improve acne classification, monitoring of treatment, and pathophysiologic characterization. In the near future, dermoscopy, RCM, and OCT could become routinely used for the evaluation of acne vulgaris to provide a deeper knowledge of the disease and to guide the clinician in the prescription of tailored treatment protocols based on each patient’s characteristics.

## 1. Introduction

Acne vulgaris is a skin disease of the pilosebaceous unit, characterized by hyperkeratinization of the infundibulum and the development of multiple inflammatory and noninflammatory lesions. Noninvasive imaging techniques have recently outlined precise microscopic features of acne elementary lesions and accurate quantifications for disease severity staging and therapeutical efficacy follow-up (Table 1) [1].

Dermoscopy is a noninvasive imaging technique that allows the identification of several features in the epidermis and dermis that are not detectable to the naked eye. It is also known as epiluminescence light microscopy and was previously known as dermatoscopy. Dermoscopy utilizes a ×10 to ×100 microscope objective with a light source to magnify and visualize structures present below the skin’s surface, such as melanin and blood vessels [2]. There are three types of dermoscopy: conventional nonpolarized dermoscopy, nonpolarized contact dermoscopy, and polarized contact dermoscopy. Many dermoscopic structures have a high degree of correspondence with pathognomonic histopathologic features, allowing a significant increase of accuracy for the diagnosis of melanoma and other skin tumors with respect to naked eye examination [2].

Reflectance confocal microscopy (RCM) acquires en-face images of the epidermis and the upper dermis, with a resolution comparable to that of histology [3]. It utilizes an 830 nm laser to capture horizontal en-face images of the skin with high resolution. The RCM imaging technique is based on the principle that different skin structures harbor different refractive proprieties, in order to generate black-and-white images. For instance, keratin, melanin, and collagen appear brighter because they have highly refractive indices, while other skin components appear darker, because of their low refractive index. The commercially available RCM devices allow for fast and reliable acquisitions of digital horizontal skin sections, with a resolution of 0.5–1 µm.

Optical coherence tomography (OCT) utilizes light backscatter from infrared light to noninvasively image the epidermis and the dermis (up to a depth of up to 1.5 mm). It provides cross-sectional 2D and 3D skin images, and depending upon the device utilized, can supply additional blood-flow information [4]. OCT is well established in noninvasive diagnostics, especially for epithelial skin tumors, enabling the visualization of architectural skin changes, but is without high cellular resolution. There are different types of OCT devices available, including frequency-domain OCT (FD-OCT), or conventional OCT, high-definition OCT (HD-OCT), and dynamic OCT (D-OCT). Recently, line-field confocal OCT (LC-OCT), which is based on the echo-time delay and amplitude of light backscattered from cutaneous microstructures through low-coherence interferometry associated with confocal spatial filtering, was implemented for the noninvasive imaging of skin lesions with very promising results [5].

The aim of this review is to systematically describe current applications of dermoscopy, RCM, and OCT in order to enhance our understanding of acne vulgaris and to improve its management.

## 2. Methods

### 2.1. Study Selection Criteria

The study followed the Preferred Reporting Items for Systematic Reviews and Meta-analyses (PRISMA) guidelines [6]. We included studies conducted on human subjects with elementary lesions of acne vulgaris, reporting assessment of the lesions with dermoscopy, RCM, and/or OCT noninvasive diagnostic tools. Studies published in any language other than English were excluded. Further criteria specified the exclusion of case reports, studies on non-human subjects, and imaging of non-elementary (e.g., scarring) lesions.

### 2.2. Data Search

The MEDLINE (PubMed) and Cochrane library electronic databases were systematically searched using the following search terms: “acne” AND (“dermoscopy” OR “reflectance confocal microscopy” OR “optical coherence tomography”). There was no limit to the search in terms of publication date, and the most recent search was run in January 2022. A manual search of reference lists was also performed.

### 2.3. Study Selection and Data Collection

Two authors independently reviewed study abstracts for inclusion and exclusion criteria (AA and AS). The following data were extracted from each of the included studies: type of study, imaging device used, number of acquired skin areas, number of patients, acne severity, aim of the study, type of treatment (Table 2).

### 2.4. Study Outcomes

The current study was designed to summarize elementary acne lesion features as observed with noninvasive techniques, including dermoscopy, RCM, and OCT. Secondary outcomes included a summary of acne pathophysiology and acne treatment monitoring.

## 3. Results

### Study Selection

**Figure 1:**
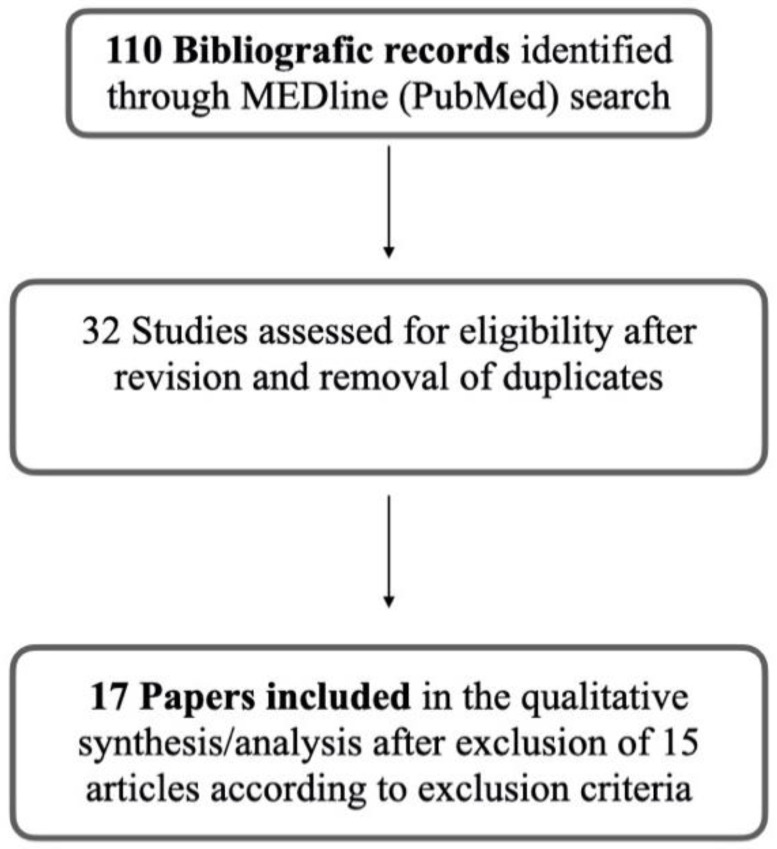
Study selection synthesis.

Following the removal of irrelevant references, the initial database search identified 110 bibliographic records. Study titles and abstracts were reviewed, and duplicates were removed; 78 were excluded. Full texts of 32 studies were assessed for further eligibility. Fifteen articles were excluded according to exclusion criteria. Finally, 17 papers were included in the qualitative synthesis/analysis (Figure 1).

## 4. Dermoscopy in Acne Lesions

Dermoscopy is a noninvasive, universally employed technique that allows the identification of several features of the epidermis and dermis that are not detectable to the naked eye. Even if dermoscopy has been essentially applied for the diagnosis and differentiation of skin tumors, recent studies have demonstrated further applications in the identification and monitoring of several inflammatory skin diseases [7,8].

**Figure 2:**
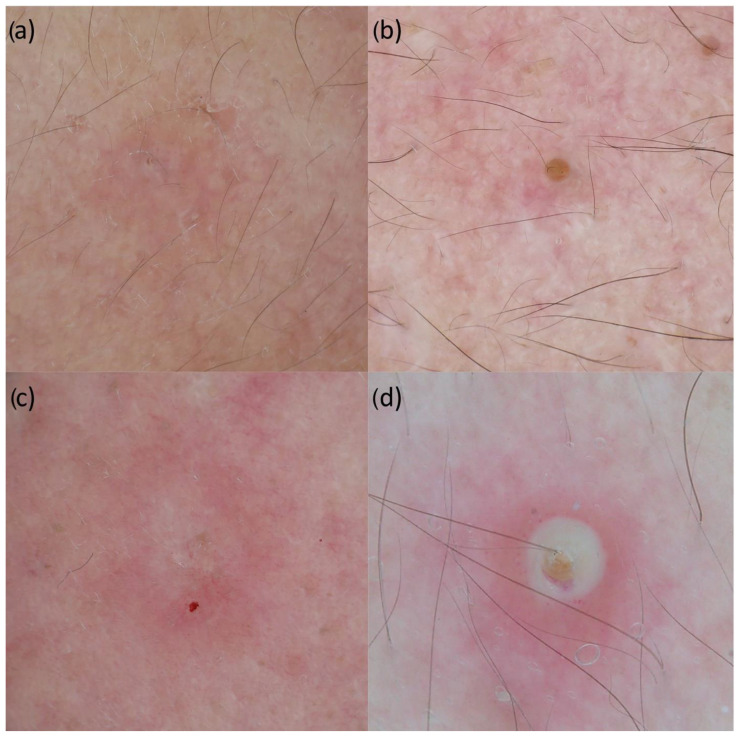
Dermoscopic images of closed comedo (**a**), open comedo (**b**), papule (**c**), and pustule (**d**).

Our search identified two manuscripts reporting dermoscopy features of acne patients [7,9]. With dermoscopy, typical acne elementary lesion features are described and defined with comedos, which appear as dilated follicles filled with a white-yellow circle or a brown plug that represents oxidized keratin, papules, which appear as erythematous roundish lesions, as a result of local inflammation, and pustules, which are centered by a white-yellowish area that corresponds to the collection of purulent material inside the cavity of the lesion (Figure 2a–d). Our search did not identify any description of peculiar vascular pattern in any of the included elementary lesions.

## 5. Rcm in Acne Lesions and Perilesional Skin

RCM is noninvasive imaging technique that allows the visualization of microscopic architectural features and individual cells of the epidermis and upper dermis at nearly histologic resolution. The histologic correspondence of structures seen using both dermoscopy and in vivo RCM has been widely demonstrated [10,11]. The use of in vivo RCM has rapidly increased over recent years because of the feasibility of the instrument and the possibility of having a more accurate presurgical diagnosis for different skin tumors, resulting in a demonstrated improvement in the diagnostic accuracy [12,13,14]

**Figure 3:**
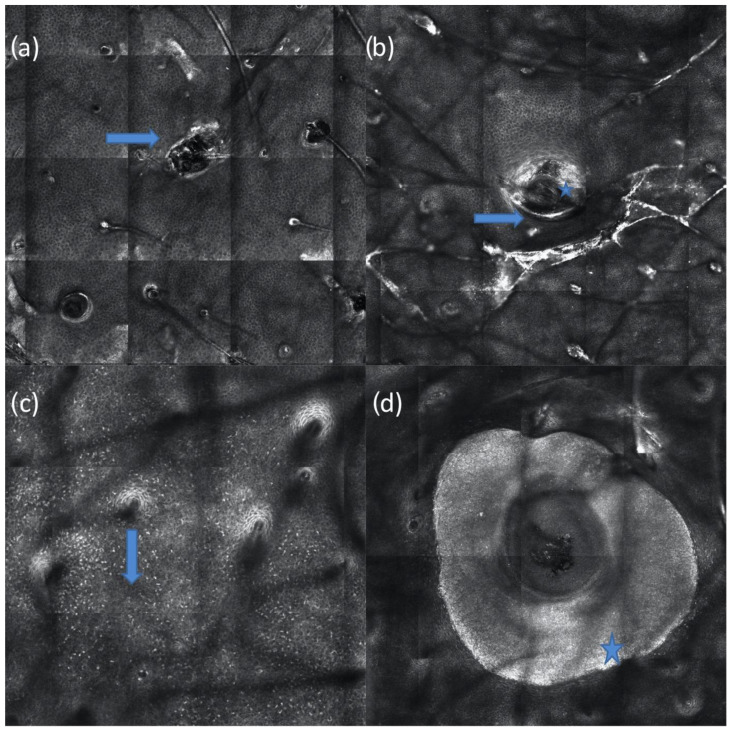
RCM images of a closed comedo (arrow), with well-defined infundibula and thick bright borders (**a**); an open comedo (arrow), with large infundibula and hyper-reflecting irregular borders filled by amorphous material (star) (**b**); a papule, showing an abundant inflammatory infiltrate composed of many sparse hyper-reflecting cells (arrow) at the spinous layer of the epidermis (**c**), and a pustule characterized by the presence of a hyper-reflecting organized inflammatory infiltrate inside the pustular cavity (star) (**d**).

We identified three studies that describe the microscopic changes observed with RCM of acne lesions [15,16,17]. Comedos are described as being characterized by enlarged infundibula (compared to follicles of normal skin), with a hyperkeratotic bright border. In detail, closed comedones present well-defined infundibula, with thick bright borders, while open comedos present larger infundibula with irregular borders that are filled by amorphous keratinized material containing multiple bright dots. Noninflamed comedos usually do not show any inflammatory infiltrate or increased vascularity in the adjacent epidermis (Figure 3a,b).

Papules are characterized by a variable RCM appearance, with abundant inflammatory phenomena, ill-delimitated borders, increased vascularity, and intact or excoriated epidermis, showing a central dark area with necrotic content and detached cellular debris (Figure 3c).

Pustules are characterized by the presence of single or multiple dark cavities filled by a granular organized inflammatory infiltrate that appear brighter than the surrounding skin. They are characterized by an abundant inflammatory infiltrate in the perilesional skin with increased vascularization in the adjacent upper dermis (Figure 3d).

In perilesional uninvolved skin, pilosebaceous units often present distinctive RCM features compared to non-acne patients. The studies report a variable amount of amorphous material inside the infundibular opening, a larger diameter, and a brighter border. These altered pilosebaceous units, which have been previously identified as microcomedos, represent the precursor lesions of acne [16].

Most studies of acne are performed on adolescent acne patients. One study reported RCM features of a population of adult women with acne and described the same RCM features commonly identified in adolescent patients [18]. Interestingly, the authors observed that microcomedones are more frequently observed in the mandibular region compared with the forehead, suggesting a correlation between microcomedones and adult acne lesions, and that pilosebaceous unit alterations are probably influenced by the topological anatomy of the face of adult women [19].

Fuchs et al. demonstrated that acne severity was positively correlated to RCM and OCT features of lesional and perilesional acne skin [15]. Higher severity grades of acne were associated with the increased detection of enlarged infundibula, hyperkeratosis, and hyperreflective keratinous content. In addition, the amount of the inflammatory phenomena was more intense in severe acne, demonstrating that a strong correlation exists between the phlogistic skin status and acne severity.

## 6. Oct in Acne Lesions

OCT is a well-established noninvasive diagnostic device, with a lateral resolution in the 3–15 µm range and a penetration depth of 1–2 mm. Dynamic OCT (D-OCT) allows the visualization of the epidermis and dermis and of the relative microvascular changes in blood flow, in order to allow the morphological and functional characterization of skin lesions [20,21,22].

**Figure 4:**
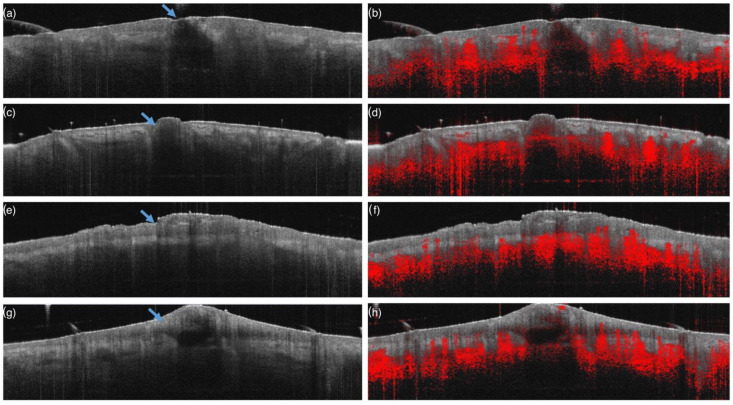
D-OCT images of a closed comedo (arrow) with the typical reverse-v-shaped morphology (**a**) showing the presence of significant vascular signal (red color) in dermis mainly at the border but not at the core of the lesion (**b**); open comedo (arrow), which is characterized by a rectangular hypoechogenic structure, with a large and thick hyper-intense plug at the top (**c**) showing the presence of significant vascular signal (red color) in dermis mainly at the border but not at the core of the lesion (**d**); papule (arrow), which is a dome-shaped lesion characterized by a diffuse inflammatory infiltrate at the epidermis and dermis (**e**) showing a prominent vascular signal (red color) in dermis both at the border and at the core of the lesion (**f**); pustule (arrow), which is a dome-shaped lesion with multiple central oval cavities containing hyper-intense inflammatory aggregates (**g**) showing enhanced vascular signal (red color) in dermis both at the border and at the core of the lesion (**h**).

Our search identified three studies that define the microscopic changes that characterize OCT images of acne lesions [15,23,24]. Comedos usually show well-defined borders and a variable darker appearance depending upon on the amount of hyperkeratinization. Closed comedos present a smaller infundibular opening and a reverse-V-shaped morphology. Sometimes, hyperechogenic structures may be found, corresponding to enlarged sebaceous glands. Open comedos are characterized by a more superficial hyperkeratinized component, with a rectangular shape and a superficial keratinized plug of variable diameters. Digital OCT (D-OCT) shows the presence of a normal vascularization in the dermis adjacent to non-inflamed comedos and an increase of vascular signal in proximity to inflamed comedos. Open comedos are characterized by a rectangular hypoechogenic structure at their boundaries with a large and thick hyper-intense plug at the top of the infundibulum. The vascular component is the same as that of closed comedos without any vascular signal in correspondence to the core of the lesion (Figure 4a–d).

Papules appear as well-defined, dark gray, dome-shaped structures with an excoriated or intact epidermis. The rete ridge is often flattened in the central part of the lesion and a hyperechogenic component in the superficial dermis is often observed. The vascular network appears prominent both in the surrounding dermis and near the core of the lesion (Figure 4e,f).

Pustules appear as well-defined, dome-shaped structures with multiple central oval cavities containing hyper-intense material inside the epidermis and the dermis. The vascular network is prominent in the dermis adjacent to the lesion. Reconstructed vascular flow is often very close to the dermal core of the pustule (Figure 4g,h).

In acne patients’ clinically uninvolved skin, there does not seem to be a remarkable difference in morphology or blood flow, compared with healthy subjects [23]. Fuchs et al. demonstrated that D-OCT blood flow was significantly increased in Investigator Global Assessment (IGA) 3 compared to IGA 1 acne patients, highlighting that blood flow parameters may be useful for the grading of acne severity [25].

## 7. Acne Pathophysiology

RCM and OCT are noninvasive diagnostic tools that can help clinicians to better understand the pathophysiology of acne. Several studies have been performed, analyzing lesional and nonlesional skin in acne patients and healthy controls. Fuchs et al. found that lesional and nonlesional skin of acne patients was characterized by an increased presence of hyperkeratinized follicles and by inflammatory changes that were positively correlated with disease severity compared with healthy controls [15].

Our group recently analyzed the progression of acne skin from the absence to the appearance and resolution of an inflammatory acne lesion [19]. Morphologic and functional data allowed the generation of an acne disease progression model characterized by the subsequent steps:

(1) Initially, acne skin is characterized by an increased number of small bright follicles (microcomedos).
(2) Subsequently, several large bright follicles appear in the area of interest, associated with increased hyperkeratinization of the infundibula, leading to its occlusion (comedos).
(3) Abruptly, several inflammatory phenomena occur with the appearance of papules, pustules, or nodular lesions with increased vessel density, exocytosis, and organized inflammatory infiltrate and an increase of adjacent small bright follicles.
(4) Finally, resolution of the inflammatory lesion is characterized by the absence of inflammatory phenomena and the persistence of an increased number of small and large bright follicles associated with dilated sebaceous gland morphology.

## 8. Noninvasive Acne Therapy Monitoring

Cutaneous noninvasive techniques represent a useful tool to obtain an objective reproducible evaluation of morphological changes within the skin, following pharmacological or other treatments.

Manfredini et al. [17] evaluated acne topical therapy efficacy using a combined method of clinical and RCM evaluation, showing that a clinical improvement was associated with a reduction of hyperkeratinized follicles, a decrease of inflammatory phenomena, and an increase of normal-appearing follicles at the end of treatment.

Fuchs et al. [26] combined in vivo RCM and OCT imaging on a total of 14 patients with acne severity IGA 0–3 to investigate trans-follicular delivery of gold microparticles (GMPs). This study demonstrated that RCM and OCT reliably visualize delivery of topically allied GMPs and that RCM detected laser-mediated thermal effects following GMP application.

Rossi et al. [27] described the first two acne patients treated with plasma exeresis using clinical picture and RCM images to objectively assess the microscopic ad macroscopic changes induced by the treatment, showing an almost complete resolution of skin lesions.

Garofalo et al. [28] applied RCM as a tool to establish the role of benzoylperoxide 4%, retinol 0.5%, mandelic acid 1%, and lactobionic acid 1% for the treatment of mild facial acne in 20 patients. They observed a remarkable reduction (>65%) of comedonic, papular and pustular lesions following treatment. These results were confirmed by RCM showing a reduction of dermal inflammation and exocytosis, and improvement of infundibular hyperkeratinization of target lesions.

Villani et al. [29] evaluated the efficacy of a gel formulation containing retinol encapsulated in glycospheres and hydroxypinacolone retinoate, an antimicrobial peptide, salicylic acid, glycolic acid, and niacinamide in 20 patients with mild acne. RCM confirmed that inflammation was reduced and infundibular hyperkeratinization improved following treatment.

Capitanio et al. [30] recruited 18 patients with mild comedonal acne in order to study the effects of RetinSphere-vit E formulation treatment. RCM highlighted the reduction of hyperkeratinization of infundibula and inflammation after treatment.

RCM and OCT imaging have also been used by Fuchs et al. [31] as a monitoring tool to assess the efficacy of adapalene-benzoyl peroxide. RCM showed a reduction of hyperkeratinization of follicular borders and intrafollicular content. OCT showed an increase of epidermal thickness that was associated with an improvement of skin trophism.

Finally, Manfredini et al. [23] demonstrated that oral antibiotic treatment improved morphologic features and decreased the vascular signal over time, as shown by OCT imaging.

## 9. Conclusions

Clinical examination represents the first line in acne diagnosis and treatment. However, dermoscopy, RCM, and OCT are further tools that can improve acne classification, monitoring of treatment, and pathophysiologic characterization.

At present there are few studies regarding acne and noninvasive imaging techniques, representing the main limitation of this review. Dermoscopy is a straightforward tool to evaluate the patient, helpful in the first approach to many diseases, routinely used in adjunct to clinic evaluation. RCM has proved useful in the evaluation of the microscopic pilosebaceous alteration at the early stages of the disease and to differentiate acne from other diseases, such as demodicosis. D-OCT enables the study of skin morphology and dermal blood flow, allowing a functional and structural characterization of acne inflammatory lesions.

To conclude, dermoscopy, RCM, and OCT give important data regarding acne lesions and patients’ skin characteristics, which should be integrated with clinical information to identify the best treatment and management possible. Noninvasive imaging devices can help clinicians to detect early new lesions, detecting ultrastructural changes and monitoring microscopic alterations, to improve treatment timing and effectiveness.

In the near future, dermoscopy, RCM, and OCT could become routinely used for the evaluation of acne vulgaris to provide a deeper knowledge of the disease and to guide the clinician in the prescription of tailored treatment protocols based on each patient’s characteristics.

## Figures and Tables

**Table 1:** Main dermoscopy, reflectance confocal microscopy, and optical coherence tomography and their histopathologic correlation in acne elementary lesions.

Elementary Acne Lesion Features	Dermoscopy Features	RCM Features	OCT Features	Histopathology Features
Comedos	Dilated pilosebaceous units filled with a white-yellow circle or a brown plug	Enlarged infundibula with a hyperkeratotic bright border	Enlarged infundibula with hyperkeratinized borders and a variable darker appearance depending on the amount of keratin.	A comedo is an altered pilo-sebaceous unit characterized by the presence of hyperkeratinization at the infundibulum and the istmus. The histopathologic appearance of comedos is characterized by the presence of a cyst-like cavity with a keratinous mass, colonized by bacteria.
Papules	Erythematous roundish lesions	Poorly defined lesions with intact or excoriated epidermis and an abundant inflammatory infiltrate in the epidermis or in the dermis.	Dome-shaped lesions with an either intact or thin and uneven epidermal surface and abundant inflammatory phenomena.	Papules are dome-shaped skin lesions characterized by the accumulation of inflammatory cells in dermis in a circumscribed area around one or more pilosebaceous units
Pustules	Inflammatory lesions centered by a white-yellowish area	Inflammatory lesions characterized by the accumulation of an organized inflammatory infiltrate and increased vascularity	Dome- shaped lesions characterized by abundant inflammatory phenomena associated to the presence of purulent aggregates inside the pustular cavity.	Pustules are dome-shaped skin lesions characterized by the accumulation of neutrophils within comedones, usually leading to the rupture of the original cystic cavity in the dermis.

**Table 2:** Data collection synthesis. (n.s., nonspecified).

Author	Number of Patients	Study Design	Acne Severity Grade	Technique	Number of Acquired Skin Areas	Aim of the Study	Type of Treatment
Manfredini 2015 [5]	35	Observational study	Absent to moderate	RCM	55	Acne characterization	n.s.
Manfredini 2019 [6]	10	Observational pilot study	Mild to moderate	RCM and OCT	70 RCM and 70 OCT	Acne characterization	n.s.
Fuchs 2018 [7]	21	Explorative	Absent to moderate	RCM and OCT	108 RCM and 54 OCT	Acne characterization	n.s.
Guenot 2018 [8]	42	Prospective	n.s.	RCM	42	Acne characterization in adult women	n.s.
Manfredini 2017 [9]	19	Observational study	Mild to moderate	RCM	76	Treatment monitoring	Hydroypinacolone retinoate/BIOPEP
Garofalo 2019 [10]	20	Pilot study	Mild	RCM	60	Treatment monitoring	Benzoylperoxide 4%, retinol 0.5%, mandelic acid 1%, and lactobionic acid 1%
Fuchs 2021 [11]	15	Prospective study	Mild to moderate	RCM and OCT	60 RCM and 60 OCT	Treatment monitoring	Adapalen-benzoyl peroxide
Manfredini 2017 [12]	31	Prospective	Mild to moderate	OCT	132	Treatment monitoring	Oral antibiotic tratment
Capitanio 2014 [13]	28	Prospective	Absent to mild	RCM	8	Treatment monitoring	Mixed RetinSphere- vitamin E formulation
Fuchs 2019 [14]	12	n.s.	Absent to moderate	RCM and OCT	109 RCM and 120 OCT	Treatment monitoring	Gold microparticles
Rossi 2018 [15]	2	n.s.	n.s.	RCM	4	Treatment monitoring	Plasma exeresis

## Data Availability

Not applicable.

## References

[1] Ulrich M., Themstrup L., De Carvalho N., Manfredi M., Grana C., Ciardo S., Kästle R., Holmes J., Whitehead R., Jemec G.B.E. (2016). Dynamic Optical Coherence Tomography in Dermatology. Dermatology.

[2] Srivastava R., Manfredini M., Rao B.K. (2019). Noninvasive imaging tools in dermatology. Cutis.

[3] Rajadhyaksha M., González S., Zavislan J.M., Anderson R.R., Webb R.H. (1999). In Vivo Confocal Scanning Laser Microscopy of Human Skin II: Advances in Instrumentation and Comparison with Histology. J. Investig. Dermatol..

[4] Sattler E., Kästle R., Welzel J. (2013). Optical coherence tomography in dermatology. J. Biomed. Opt..

[5] Ruini C., Schuh S., Sattler E., Welzel J. (2020). Line-field confocal optical coherence tomography—Practical applications in dermatology and comparison with established imaging methods. Ski. Res. Technol..

[6] Page M.J., McKenzie J.E., Bossuyt P.M., Boutron I., Hoffmann T.C., Mulrow C.D., Shamseer L., Tetzlaff J.M., Akl E.A., Brennan S.E. (2021). The PRISMA 2020 statement: An updated guideline for reporting systematic reviews. Int. J. Surg..

[7] Lacarrubba F., Ardigò M., Di Stefani A., Verzì A.E., Micali G. (2018). Dermatoscopy and Reflectance Confocal Microscopy Correlations in Nonmelanocytic Disorders. Dermatol. Clin..

[8] Guida S., Longo C., Casari A., Ciardo S., Manfredini M., Reggiani C., Pellacani G., Farnetani F. (2015). Update on the use of confocal microscopy in melanoma and non-melanoma skin cancer. G. Ital. Dermatol. Venereol..

[9] Alfaro-Castellón P., Mejía-Rodríguez S.A., Valencia-Herrera A., Ramírez S., Mena-Cedillos C. (2012). Dermoscopy Distinction of Eruptive Vellus Hair Cysts with Molluscum Contagiosum and Acne Lesions. Pediatr. Dermatol..

[10] Pellacani G., Longo C., Malvehy J., Puig S., Carrera C., Segura S., Bassoli S., Seidenari S. (2008). In Vivo Confocal Microscopic and Histopathologic Correlations of Dermoscopic Features in 202 Melanocytic Lesions. Arch. Dermatol..

[11] Farnetani F., Manfredini M., Chester J., Ciardo S., Gonzalez S., Pellacani G. (2019). Reflectance confocal microscopy in the diagnosis of pigmented macules of the face: Differential diagnosis and margin definition. Photochem. Photobiol. Sci..

[12] González S., Tannous Z. (2002). Real-time, in vivo confocal reflectance microscopy of basal cell carcinoma. J. Am. Acad. Dermatol..

[13] Boone M., Jemec G.B.E., Del Marmol V. (2012). High-definition optical coherence tomography enables visualization of individual cells in healthy skin: Comparison to reflectance confocal microscopy. Exp. Dermatol..

[14] Ahlgrimm-Siess V., Horn M., Koller S., Ludwig R., Gerger A., Hofmann-Wellenhof R. (2009). Monitoring efficacy of cryotherapy for superficial basal cell carcinomas with in vivo reflectance confocal microscopy: A preliminary study. J. Dermatol. Sci..

[15] Fuchs C.S.K., Andersen A.J.B., Ardigo M., Philipsen P.A., Haedersdal M., Mogensen M. (2018). Acne vulgaris severity graded by in vivo reflectance confocal microscopy and optical coherence tomography: In vivo rcm and oct imaging of acne morphology. Lasers Surg. Med..

[16] Manfredini M., Mazzaglia G., Ciardo S., Farnetani F., Mandel V.D., Longo C., Zauli S., Bettoli V., Virgili A., Pellacani G. (2014). Acne: In vivo morphologic study of lesions and surrounding skin by means of reflectance confocal microscopy. J. Eur. Acad. Dermatol. Venereol..

[17] Manfredini M., Greco M., Farnetani F., Mazzaglia G., Ciardo S., Bettoli V., Virgili A., Pellacani G. (2016). In vivo monitoring of topical therapy for acne with reflectance confocal microscopy. Ski. Res. Technol..

[18] Guenot L.M., Jourdain M.V., Saint-Jean M., Corvec S., Gaultier A., Khammari A., Le Moigne M., Boisrobert A., Paugam C., Dréno B. (2018). Confocal microscopy in adult women with acne. Int. J. Dermatol..

[19] Manfredini M., Bettoli V., Sacripanti G., Farnetani F., Bigi L., Puviani M., Corazza M., Pellacani G. (2019). The evolution of healthy skin to acne lesions: A longitudinal, in vivo evaluation with reflectance confocal microscopy and optical coherence tomography. J. Eur. Acad. Dermatol. Venereol..

[20] Mogensen M., Thrane L., Jørgensen T.M., Andersen P.E., Jemec G.B.E. (2009). OCT imaging of skin cancer and other dermatological diseases. J. Biophotonics.

[21] Sattler E.C.E., Poloczek K., Kästle R., Welzel J. (2013). Confocal laser scanning microscopy and optical coherence tomography for the evaluation of the kinetics and quantification of wound healing after fractional laser therapy. J. Am. Acad. Dermatol..

[22] Manfredini M., Chello C., Ciardo S., Guida S., Chester J., Lasagni C., Bigi L., Farnetani F., Bettoli V., Pellacani G. (2022). Hidradenitis Suppurativa: Morphologic and vascular study of nodular inflammatory lesions by means of optical coherence tomography. Exp. Dermatol..

[23] Manfredini M., Greco M., Farnetani F., Ciardo S., De Carvalho N., Mandel V.D., Starace M., Pellacani G. (2017). Acne: Morphologic and vascular study of lesions and surrounding skin by means of optical coherence tomography. J. Eur. Acad. Dermatol. Venereol..

[24] Hermsmeier M., Sawant T., Chowdhury K., Nagavarapu U., Chan K.F. (2019). First Use of Optical Coherence Tomography on In Vivo Inflammatory Acne-Like Lesions: A Murine Model. Lasers Surg. Med..

[25] Manfredini M., Liberati S., Ciardo S., Bonzano L., Guanti M., Chester J., Kaleci S., Pellacani G. (2020). Microscopic and functional changes observed with dynamic optical coherence tomography for severe refractory atopic dermatitis treated with dupilumab. Ski. Res. Technol..

[26] Fuchs C.S.K., Ortner V.K., Mogensen M., Philipsen P.A., Haedersdal M. (2019). Transfollicular delivery of gold microparticles in healthy skin and acne vulgaris, assessed by in vivo reflectance confocal microscopy and optical coherence tomography. Lasers Surg. Med..

[27] Rossi E., Mandel V.D., Paganelli A., Farnetani F., Pellacani G. (2018). Plasma exeresis for active acne vulgaris: Clinical and in vivo microscopic documentation of treatment efficacy by means of reflectance confocal microscopy. Ski. Res. Technol..

[28] Garofalo V., Cannizzaro M.V., Mazzilli S., Bianchi L., Campione E. (2019). Clinical evidence on the efficacy and tolerability of a topical medical device containing benzoylperoxide 4%, retinol 0.5%, mandelic acid 1% and lactobionic acid 1% in the treatment of mild facial acne: An open label pilot study. Clin. Cosmet. Investig. Dermatol..

[29] Villani A., Annunziata M.C., Cinelli E., Donnarumma M., Milani M., Fabbrocini G. (2020). Efficacy and Safety of a New Topical Gel Formulation Containing Retinol Encapsulated in Glycospheres and Hydroxypinacolone Retinoate, an Antimicrobial Pep-tide, Salicylic Acid, Glycolic Acid and Niacinamide for the Treatment of Mild Acne: Preliminary Results of a 2-Month Prospective Study. G. Ital. Dermatol. Venereol..

[30] Capitanio B., Lora V., Ludovici M., Sinagra J.L.M., Ottaviani M., Mastrofrancesco A., Ardigo M., Camera E. (2014). Modulation of sebum oxidation and interleukin-1? levels associates with clinical improvement of mild comedonal acne. J. Eur. Acad. Dermatol. Venereol..

[31] Fuchs C.S.K., Ortner V.K., Hansen F.S., Philipsen P.A., Haedersdal M. (2021). Subclinical effects of adapalene-benzoyl peroxide: A prospective in vivo imaging study on acne micromorphology and transfollicular delivery. J. Eur. Acad. Dermatol. Venereol..

